# Is Climate Change Slowing the Urban Escalator Out of Poverty? Evidence from Chile, Colombia, and Indonesia

**DOI:** 10.3390/ijerph20064865

**Published:** 2023-03-10

**Authors:** Shohei Nakamura, Kseniya Abanokova, Hai-Anh H. Dang, Shinya Takamatsu, Chunchen Pei, Dilou Prospere

**Affiliations:** 1 World Bank, Washington, DC 20433, USA; 2 International School, Vietnam National University, Hanoi 123105, Vietnam; 3 Indiana University, Bloomington, IN 47405, USA; 4 IZA, Schaumburg-Lippe-Strasse 5-9, 53113 Bonn, Germany; 5 School of Economics and Resource Management, Beijing Normal University, Beijing 100875, China; 6 Osaka School of International Public Policy, Osaka University, Osaka 560-0021, Japan

**Keywords:** migration, urban agglomeration, poverty, climatic change, flooding

## Abstract

While urbanization has great potential to facilitate poverty reduction, climate shocks represent a looming threat to such upward mobility. This paper empirically analyzes the effects of climatic risks on the function of urban agglomerations to support poor households’ escape from poverty. Combining household surveys with climatic datasets, our analyses of Chile, Colombia, and Indonesia find that households in large metropolitan areas are more likely to escape from poverty, indicating better access to economic opportunities in those areas. However, climate shocks such as extreme rainfalls and high flood risks significantly reduce upward mobility, thus offsetting such benefits of urban agglomerations. The findings underscore the need to enhance resilience among the urban poor to allow them to fully utilize the benefits of urban agglomerations.

## 1. Introduction

Urban areas around the world are attractive places for people looking for opportunities for a better life. About 55 percent of the world’s population lived in urban areas in 2018 and that number is likely to grow by 68 percent by 2050 [1]. Urban agglomerations spur economic growth through productivity gains within economic sectors and structural transformation [2,3,4,5]. At the same time, urban residents tend to be vulnerable to climatic and environmental shocks triggered by increased economic activities and they are often pushed back into, or remain trapped in, poverty [6]. Without proper mitigation and adaptation measures against climate change, the benefits of urbanization could be negated [7].

In this context, this paper attempts to test the following two hypotheses. The first is that people are more likely to become or stay nonpoor in larger or denser cities, compared to smaller or less densely populated towns. The second hypothesis is that large or dense cities that are more exposed to climatic and environmental shocks offer residents a lower chance of becoming or staying nonpoor, compared with cities of similar size but with less exposure to such shocks. By empirically testing those hypotheses, we investigate the following key question: Do climatic and environmental shocks hamper the key function of urban agglomerations as the escalator out of poverty in the developing world? Confirming this question is critically important as it underscores the need for policy interventions aiming at achieving inclusive and green growth through urban development.

We developed an analytical approach to investigating this research question with and without (actual) panel data by combining both panel and synthetic panel data with climatic data. The synthetic panel method is a useful approach to analyzing poverty dynamics when panel household survey datasets are not available. We developed synthetic panel datasets out of repeated cross-sectional household surveys in Chile between 2011 and 2015 and Colombia between 2008 and 2010. We then examined the relationship between poverty changes over time and city population size as well as heterogeneity of the estimation results by flood risks. We also applied a similar analytical framework to analyze another country, Indonesia. Since actual panel data are available for Indonesia, we estimated two-way fixed-effect (FE) regressions on the five waves of Indonesia Family and Life Surveys (IFLS) to analyze the variation of probabilities of poor households escaping from poverty by urban agglomeration classifications and climatic shocks/risks. The IFLS spans from 1993 to 2014 over 298 districts and tracks the same households over time. We focused on flood as the climatic factor by measuring the rainfall anomaly and heavy rain measured by the Standardized Precipitation Evapotranspiration Index (SPEI). Yet, it should be noted that FE regressions do not allow us to fully identify causal effects; thus, for caution, the estimation results should be interpreted as correlations.

Our analysis supports the hypothesis that climatic risks could undermine the upward mobility facilitated by urban agglomerations. Analysis using the synthetic panels in Chile between 2011 and 2015 and Colombia between 2008 and 2010 indicates a reduction in urban poverty rates as measured by the upper-middle-income international poverty line (US$5.5 per day in 2011 purchasing power parity [PPP]). In those countries, 7.4 percent and 4.1 percent of the urban poor escaped from poverty during the aforementioned periods, respectively. The analysis finds that the probabilities of households’ transition from poor to nonpoor status were positively correlated with the city population size in both countries. More importantly, such upward mobility was observed only in larger cities with low flood risk. The FE results for Indonesia suggest that, compared with rural areas, the chance of getting out of vulnerability is higher by 7.0 percentage points in metropolitan cores. However, heavy rainfall and high flood risk decrease upward mobility in the cores and urban peripheries of metropolitan areas.

Our paper contributes to the literature on the nexus between urbanization and poverty. Several studies show urban-rural gaps in productivity, wages, and amenities [see [8] for a review]. Some studies find earnings and welfare gains from rural to urban migration in poorer countries, such as rural to urban migration in Tanzania [9], seasonal migration in Bangladesh [10], and the interplay of locations and migrant characteristics in determining gains in China [11]. Hamory et al. [12] analyzed panel datasets in Indonesia and Kenya, finding that a large part of the measured returns from migration came from the sorting of migrants. However, very few studies have analyzed the role of climate change as a hindrance to urban agglomeration as the urban escalator out of poverty. Therefore, we attempt to shed light on this mostly unexplored topic.

## 2. Framework

### 2.1. Urban Escalator Out of Poverty: Hypothesis 1

**Figure 1:**
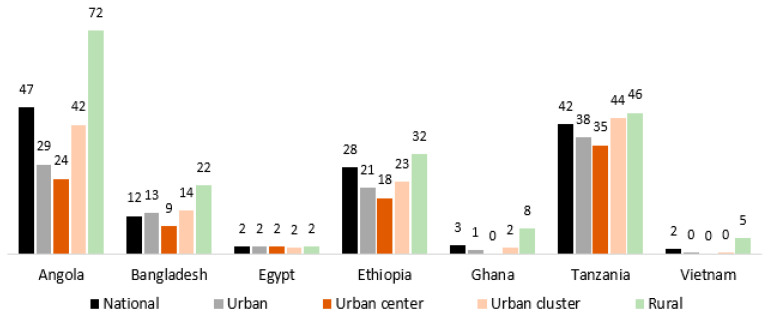
Subnational extreme poverty rates (percent) across countries; Source: Combes et al. [18]; Note: Poverty is measured with the international poverty line (US$1.9 in 2011 PPP). Following the Degree of Urbanization methodology [25], urban centers (clusters) are defined based on spatially contiguous sets of 1 km^2^ grid cells for which population density of each cell ≥ 1500 (300) people per km^2^ and aggregate settlement population ≥ 50,000 (5000).

Larger or denser urban areas potentially provide people with ample economic opportunities to escape from poverty through better access to higher-wage jobs, higher-quality infrastructure, services, and so on. More urbanized areas also tend to be characterized by higher incomes and consumption, higher productivity, better access to services, and higher human capital. Earlier studies have found that nominal wages are higher in larger or denser cities due to productivity gains from agglomeration economies in both richer countries [13,14,15,16] and poorer countries [17,18,19,20]. On the other hand, some studies highlight urbanization without growth [21,22,23,24]. Rural to urban migration can result in higher levels of welfare, given the gaps in income and amenities in the developing world [8]. When both poverty and urban areas are measured in a comparable way across countries, poverty tends to be lower in dense urban areas, as shown in Figure 1 below that is based on [18]. 

Nevertheless, the contribution of urbanization to poverty reduction is not self-evident. Larger or denser cities do not necessarily help people escape from poverty, given the various challenges of overcrowding, traffic congestion, crime, air pollution, high cost of living, lack of jobs for low-skilled workers, housing segregation, and so on. Additionally, a cross-sectional negative correlation between population density and poverty by itself could simply be an indication of a linear relationship and does not necessarily indicate upward mobility. Thus, it is not evident, a priori, that larger or denser cities are the best places for people to escape from poverty (see, for example, the argument favoring secondary towns in [26,27]).

Therefore, it is an empirical question whether urban agglomerations facilitate poverty reduction. The first hypothesis to be tested is that people are more likely to become or stay nonpoor in larger or denser cities, compared with smaller or less densely populated towns.

### 2.2. Climatic and Environmental Stressors: Hypothesis 2

Even if urban agglomerations support poverty reduction, upward mobility of the poor could be hampered by climatic shocks and risks. Urban households may fall into poverty due to higher exposure to shocks, asset vulnerability, and lack of socioeconomic resilience [6]. In urban areas, poor households could be more exposed to environmental hazard risks, building density and overcrowdedness, and inadequate infrastructure. The more urban a location is, the scarcer and more expensive the land becomes, pushing the poor into undesirable and risky locations at the peripheries of cities. Furthermore, as food consumers (rather than producers), urban households are also vulnerable to food price shocks triggered by climate anomalies.

Indeed, studies found urban households to be more vulnerable to flooding/drought shocks in different countries. For example, [28] analyzed the impacts of a severe tropical storm that hit Guatemala in 2020 with the largest rainfall in the country during the last five decades. Median per capita consumption fell more (by 12.6 percent) in urban areas, significantly increasing urban poverty. Rising food prices due to disasters lowered urban households’ consumption, while a social safety net program protected mainly rural households.

Upward mobility offered by agglomeration economies could be hindered by climatic and environmental stressors. Therefore, we hypothesize that large or dense cities that are more exposed to climatic and environmental shocks do not offer residents a higher chance of becoming or staying nonpoor, compared with cities of smaller size.

## 3. Methodology

### 3.1. Data

A straightforward way to examine the hypotheses above is to look at poverty dynamics with panel household surveys that cover multiple time points. Such data can help remove the unobserved impacts of time-invariant household characteristics. However, due to the widespread absence of nationally representative panel household surveys in poorer countries, it would be useful to develop and test an empirical approach that is based on repeated cross-sectional household surveys. As such, we selected Chile, Colombia, and Indonesia for this study to demonstrate analysis with and without panel datasets. All these countries are (lower and upper) middle-income countries, thus our analysis can be relevant for other middle-income countries that may be more affected by climate change. In addition, while household welfare is measured by income for Chile and Colombia, it is measured by consumption expenditure for Indonesia. The setting of Indonesia—its rapid urbanization and heterogenous climatic characteristics across subnational regions—is particularly suitable to our study. Highly urbanized countries like Chile and Colombia also offer useful urban density variations for analysis.

To examine our research question and hypotheses, we combined household surveys with climatic datasets. For Chile and Colombia, we constructed synthetic panel datasets out of repeated cross-sectional household surveys. Flood risk is estimated as a key climate factor for each town. For Indonesia, we relied on panel household surveys (IFLS), combined with two climate indicators: SPEI and the flood risk index.

#### 3.1.1. Synthetic Panel Data for Chile and Colombia

Following [29,30], we applied the synthetic panel method to the household surveys of Chile (Encuesta de Caracterización Sociooeconómica Nacional [CASEN]) between 2011 and 2015 and Colombia (Gran Encuesta Integrada de Hogares [GEIH]) between 2008 and 2010. The years of the surveys for both countries were selected based on the availability of the subnational location information that can be matched with climatic layers on the geographic information system (GIS) platform.

The synthetic panel method essentially exploits the time-invariant variables in the cross-sectional surveys and some cohort-based assumptions about the error terms to construct the synthetic panels. The methodology is described in detail in Appendix B. Recent applications and further validations of the synthetic panel methods have been implemented using household survey data from various countries in Sub-Saharan Africa, East Asia and Pacific, Europe and Central Asia, Latin America, South Asia, and the Middle East and North Africa (see [31,32,33] for recent reviews).

We began by identifying the potential time-invariant variables available in two rounds of cross-sectional surveys, which included household heads’ gender, age, level of education, and residence area (that is, urban or rural). These variables can usually be assumed to be time-invariant if the underlying population remains unchanged over time. One way to test this assumption is to use a t-test for the equality of the means of the same variables in the two survey rounds. We provided test results that allow for the complex survey design in Table A1, Appendix A for Chile and Table A2, Appendix A for Colombia. The assumption of the equality of means over time is satisfied for household heads’ gender and (accomplishment of the) secondary level of education in Chile and heads’ secondary and tertiary levels of education in Colombia. The assumption is satisfied for residence areas in both countries. Although the difference in heads’ primary level of education is statistically significant for Colombia, it practically has rather similar means. The differences for heads’ primary and tertiary levels of education in Chile and heads’ gender in Colombia are less than five percentage points. Thus, these may not make much difference to the final estimates in practice. The first-stage regressions using data from the two cross-sections are shown in Table A3, Appendix A for Chile and Table A4, Appendix A for Colombia. The adjusted R^2^ for these equations range from 0.29 (Chile) to 0.35 (Colombia) which indicates a good fit.

#### 3.1.2. IFLS Panel Household Survey Data: Indonesia

The IFLS includes a total of 54,000 household observations over five waves from 2556 subdistricts in 26 provinces. Focusing on household socioeconomic and health aspects, the survey was conducted for the first time in 1993, covering 13 of the total 26 provinces in the country. In a sample of 22,000 individuals from 7224 households, the survey collected data on individual respondents and their families (households) in addition to data on communities, health, and education facilities. In 1997/98, the second wave was administered to the same respondents with a recontact rate of 94.4 percent. The third wave in 2000 managed to recontact 95.3 percent of the first wave sample while the fourth and the fifth rounds conducted in 2007/08 and 2014/15 recontacted 93.6 and 90.5 percent, respectively, of the first wave sample [34].

We measured poverty based on per capita consumption expenditures, using the national poverty line following [34]. The IFLS collects data on the consumption expenditure of 37 food items over a seven-day recall period and various nonfood items. Nonfood expenditures include household amenities (for example, refrigerator, TV, and telephone); housing; assorted items such as clothing, furniture, medical, ceremonies, education (tuition, uniform, transportation, boarding); and others. Regarding the housing expenditure, the actual monthly rent paid was recorded. However, if the household owns the house, the estimated rent was imputed. The nominal consumption aggregate is both temporally and spatially deflated. Temporal deflation is based on the consumer price index series; spatial deflator is calculated based on the ratio of the regional poverty lines to the national poverty line, obtained from the National Socioeconomic Survey (SUSENAS) of the corresponding wave.

In addition to poverty, we identified vulnerable people using a vulnerability line that is set at 1.5 times the poverty line.

Roberts et al. [35] highlight the importance of classifying urban areas based on their functionality instead of mere population size in Indonesia. Following [36], we defined the following four location categories: (1) metro core, which stands for Jakarta or districts with the highest population density for other metros; (2) urban peripheries, which are predominantly urban non-core districts; (3) other urban areas that account for single-district metro (predominantly urban with kotas) or non-metro urban (predominantly urban non-metro districts); and (4) rural areas, which encompass the rural periphery (predominantly rural non-core district) or non-metro rural areas (predominantly rural non-metro districts).

#### 3.1.3. Climate Data: Flood Risk Index and SPEI

To account for climatic and environmental shocks, we used two indicators: flood risk index and the SPEI. Those climatic variables are prepared at the subdistrict level.

The primary climatic stressor analyzed in this study is flood risk, given its potential threat to urban livelihood. To capture the flood risk, we used the flood depth data provided by FATHOM in 2016. The flood depth is expressed in meters and computed at 3 arc-second (approximately 90 m) resolution and has a global coverage between 56° S and 60° N. 

**Figure 2:**
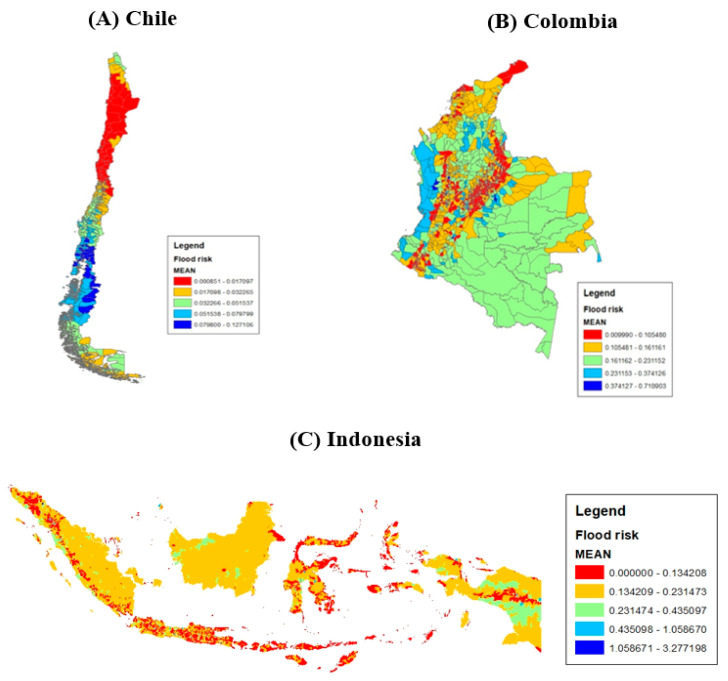
The maps of 100-year flood risks; Source: Based on FATHOM data.

The computation is based on pluvial data with a return period of 100 years, so-called 1-in-100 flood depth (See https://agupubs.onlinelibrary.wiley.com/doi/epdf/10.1002/2015WR016954 (accessed on 3 March 2023) for more details on the computation method). The 1-in-100 flood depth means that a flood event has a 1 percent probability of occurring in any given year within 100 years. We classified the areas with the top 25 percent flood depth in each country as high flood risk areas. As this is an indicator of long-term flood risk, the index essentially does not change over time. The flood risk maps for three case countries are shown in Figure 2.

Taking advantage of the long-run IFLS panel data, we additionally analyzed rainfall anomalies as a climatic factor for Indonesia. The SPEI is a multiscalar drought index [37]. The construction requires data on temperature, precipitation, and potential evaporation. Accordingly, we processed monthly precipitation and potential evapotranspiration derived from the terraclimate data from 1958 to 2020. The SPEI data are fitted to a gamma distribution and normalized to a flexible multiple time scale such as 1, 4, 6, 12, 24, and 48 months. For the study, we considered a 12-month time scale for each year from 1993 to 2015 with two lag periods for each IFLS wave. The computation involves the following three steps [38]. We first compute the difference (D) between precipitation and evapotranspiration (PET) and accounted for the climatic water balance defined at the monthly level. The Penman-Monteith equation is used to approximate the PET (as recommended by the Food and Agriculture Organization of the United Nations (FAO) as the best method for determining reference evapotranspiration). Maximum temperature, minimum temperature, vapor pressure, precipitation accumulation, downward surface shortwave radiation, and wind speed are used as input data. The next step is the aggregation of the climatic water balance at different time scales and finally, we standardize the time series according to a gamma distribution. The SPEI is then computed as the standardized values of the gamma function.

Negative SPEI values represent rainfall deficit—less than median precipitation—and high potential evapotranspiration (dry) starts when the SPEI value is equal to or below −1.0. On the other hand, positive SPEI values indicate rainfall surplus—greater than median precipitation—and low potential evapotranspiration (wet) starts when the SPEI value is equal to or above 1.0.

**Figure 3:**
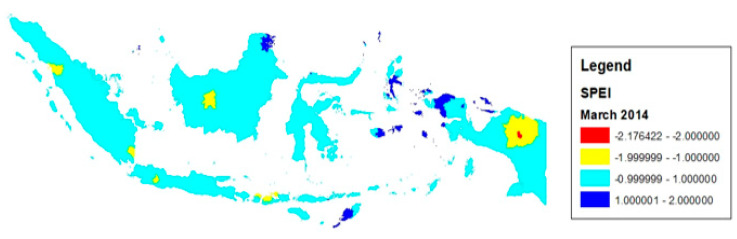
12-month SPEI in Indonesia, March 2014; Source: Based on terraclimate data.

Figure 3 plots the SPEI at the district level for Indonesia for March 2014.

#### 3.1.4. Descriptive Statistics

Table 1 presents the summary statistics for Chile (Panel A) and Colombia (Panel B) based on the synthetic panel data. Households’ upward poverty mobility (i.e., the probability of transition from being poor to nonpoor) is the outcome variable. The average upward mobility is 73.1 percent for Chile during 2011–2015 and 16.8 percent for Colombia during 2008–2010. About 24.2 and 13.8 percent of the households in Chile and Colombia respectively are in high flood risk areas.

Table 2 presents the summary statistics of key variables for Indonesia. Households’ poverty (1 = nonpoor; 0 = poor) and vulnerability (1 = neither poor nor vulnerable; 0 = poor or vulnerable) statuses are used as the (dummy) outcome variables for our regression analysis. Around 88 percent of household observations in our five-wave panel data are nonpoor, while 69 percent are neither poor nor vulnerable. The urban location typology—metro core, periphery urban, other urban, and the rural area—are also defined as dummy variables. Around 45 percent of household observations are from periphery urban, followed by other urban (19.2 percent), rural (18.6 percent), and metro core areas (17.4 percent). Households’ movements across locations between each of the five IFLS waves are summarized in Table A5, Appendix A. About 5.9 percent of households are in SPEI-dry areas, 90.7 percent are in areas with SPEI-normal, and 3.4 percent are in areas that experience heavy rains. About 25 percent of households are exposed to high flood risks.

### 3.2. Econometric Approach

We first used the synthetic panel to examine the correlational relationship that supports the first hypothesis for Colombia and Chile. To further test the second hypothesis for Colombia and Chile with synthetic panel data, we estimated the following first-difference regression model for household i in city j with the probability of transition from poor to nonpoor status between the two time points t_0_ and t_1_, or $y_{\mathrm{ij}\left(t0-t1\right)}$:

(1) $$y_{\mathrm{ij}\left(t0-t1\right)}=\alpha +\beta_{1}{\mathrm{POPSIZE}}_{j,t0}+\beta_{2}\left({\mathrm{POPSIZE}}_{j}\times {\mathrm{CLMT}}_{j}\right)+\beta_{3}{\mathrm{CLMT}}_{j}+\varepsilon_{\mathrm{ij}}$$

with ${\mathrm{POPSIZE}}_{j,t0}$ as the variable indicating the population size of city j at year t_0_ and ${\mathrm{CLMT}}_{j}$ as the 1-in-100-year flood risks at city j, $\varepsilon_{\mathrm{ijt}}$ is the error term. The parameter $\beta_{2}$ indicates how the relationship between city population size and upward mobility (that is, the probability of poor people escaping from poverty) varies by climatic risks.

To test our first hypothesis that urban areas support upward mobility for Indonesia where the panel data are available, we estimated the following two-way FE model:

(2) $$y_{\mathrm{ijt}}=\alpha +\beta_{4}{\mathrm{CITY}}_{\mathrm{ij}}+\gamma_{i}+\delta_{t}+\varphi_{\mathrm{ijt}}$$

where $y_{\mathrm{ijt}}$ stands for the poverty status (1 = nonpoor; 0 = poor) or vulnerability status (neither poor nor vulnerable = 1; 0 = poor or vulnerable) of household (i) in city (j) at year (t). Since there are no data on population size for Indonesia, we employed the variable ${\mathrm{CITY}}_{j}$ that indicates the location typology—metro core, urban periphery, other urban areas, and rural areas, with the rural areas as the reference category—for this country. $\gamma_{i}$ and $\delta_{t}$ stand for the household FEs and the year FEs, respectively. With household FEs, we focused on the probability of escaping poverty among the movers. We expected the parameter $\beta_{4}\;$for multidistrict metropolitan areas to be positive based on the first hypothesis.

For the second hypothesis, we analyzed the interaction of climatic conditions with the location effect of urban areas on poverty by adding to Equation (2) an interaction term between the location typology and the climate variable.

(3) $$y_{\mathrm{ijt}}=\alpha +\beta_{5}{\mathrm{CITY}}_{\mathrm{ij}}+\beta_{6}\left({\mathrm{CITY}}_{\mathrm{ij}}\times {\mathrm{CLMT}}_{\mathrm{jt}}\right)+\beta_{7}{\mathrm{CLMT}}_{\mathrm{jt}}+\gamma_{i}+\delta_{t}+\varphi_{\mathrm{ijt}}$$

where ${\mathrm{CLMT}}_{\mathrm{jt}}$ indicates the exposure to flood or flood risks. For exposure to flood, the precipitation anomalies were measured for each IFLS wave (see Section 3.1). The parameter $\beta_{6}$, the coefficient for the interaction term, captures the effect of the climate shocks associated with the city indicators. We estimated the panel regressions in Equations (2) and (3) as linear probability models with standard errors clustered at the enumerator areas.

## 4. Results

### 4.1. Chile and Colombia: Synthetic Panel Analysis

The results of the synthetic panel analysis for Chile and Colombia show that the probabilities of urban residents escaping poverty are positively associated with the population size of their cities. From 2011 to 2015 in Chile, 7.4 percent of the urban population (or two-thirds of the urban poor) escaped from poverty. As shown in Column 1 in Table 3, upward mobility is positively correlated with the population size of cities. A similar correlation is observed for Colombia between 2008 and 2010 (Column 1 in Table 4), where 4.1 percent of the urban population escaped from poverty.

**Figure 4:**
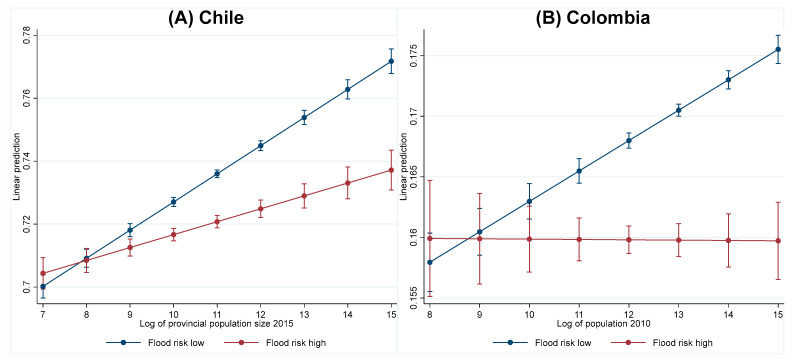
Probability of poverty transition by city population size and flood risk for Chile (**A**) and Colombia (**B**); Source: Authors’ construction.; Note: The predicted probabilities of becoming nonpoor are based on the results of Columns 4 in Table 3 (Chile) and Table 4 (Colombia). The error bars indicate 95% confidence intervals (CIs).

We then estimated Equation (1) for Chile and Colombia to examine heterogeneity of flood risks for the relationship of poverty transition and city population size. As shown in Column 4 in Table 3 and Table 4, the interaction term between the log of city population size and flood risk variables is negative (−0.005 for Chile and −0.003 for Colombia), indicating that upward mobility in large cities tends to be limited if households face high flood risks. With flat lines for high-risk areas and steep lines for low-risk areas, Figure 4 clearly shows such heterogeneity by flood risks.

### 4.2. Indonesia: Panel Data Analysis

#### 4.2.1. Urban Escalator Out of Poverty

Table 5 summarizes the estimation results of the linear probability models for Equation (2) using household nonpoor status as the dependent variable. Columns 1 and 2 report specifications without the household FEs, whereas Columns 3 and 4 include the household FEs. The year FEs are included in Columns 2 and 4. The main variable of interest is the indicator for metro core and its coefficient indicates the probability of households becoming nonpoor relative to those in rural areas.

In the baseline specification without any FEs (Column 1), the coefficient estimate for metro core is 0.065 (90% CI = 0.019), meaning that the metro core offers a 6.6 percentage point higher probability of escaping poverty in comparison with the rural areas. Other urban areas also have a positive coefficient of 0.034 (90% CI = 0.020). By contrast, peri-urban areas show a negative coefficient, indicating a lower chance of escaping poverty (compared with rural areas). Adding the year FEs in Column 2 does not significantly change the result. With the household and year FEs (Column 4), the estimated coefficient for the metro core is reduced to 0.031 (90% CI = 0.031). Other urban areas show an even smaller estimated coefficient (0.0007, 90% CI = 0.036).

The regressions in Table 6 replace the outcome variable with an indicator for vulnerability. In Columns 2 and 4, the estimated coefficient for the metro core is −0.141 (without household FEs) and −0.070 (with household FEs), respectively. That is, the probability of being neither poor nor vulnerable increases for households living in the metro core areas, regardless of whether the econometric specification is run with or without household FEs. In other words, people living in (or moving to) the metro core areas are less likely to become poor or vulnerable.

#### 4.2.2. Climatic Shock on the Urban Escalator

##### Flood Risk as a Shock Indicator

**Figure 5:**
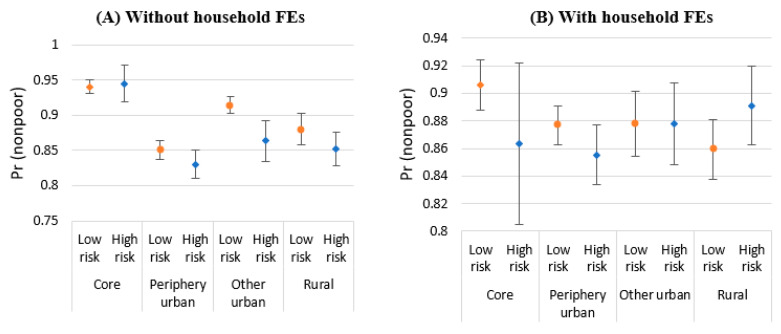
Predicted probability of being nonpoor by locations without household FEs (**A**) and with household FEs (**B**); Source: Authors’ construction.; Note: The predicted probabilities of becoming nonpoor are based on the results of Columns 2 and 4 in Table 7. Error bars indicate 90% CI.

Table 7 presents the estimates for Equation (3), showing the interaction between effects of flood risks (as a climate shock indicator) and urban location on poverty. Column 4 with the interaction between location and flood risk variables, as well as household and year FEs, shows that the probability of being nonpoor decreases due to high flood risks by 7.4 percentage points (90% CI = 0.066) in metro core and 5.33 percentage points (90% CI = 0.040) in periphery urban areas, respectively. That is, in metro areas, high flood risk lowers the chance of poverty escape in comparison with low-risk areas. The predicted probabilities in Figure 5 show that high flood risk areas have a lower predicted probability of being nonpoor for all the locations, compared with the low-risk areas, except for the metro core without household FEs and rural areas with household FEs.

##### SPEI as a Shock Indicator

We replaced flood risk with SPEI as the second climatic shock indicator in Table 8. We kept the nonpoor status as the dependent variable. As explained in Section 3, we divided the SPEI into three categories: SPEI-rainy (SPEI > 2.0), SPEI-normal (−2 > SPEI > 2), and SPEI-dry (SPEI < −2.0).

The coefficient estimate for the interaction between the metro core and SPEI-rainy variables is −0.098 (90% CI = 0.058) in Column 4 with household FEs, suggesting that metro core areas that experienced heavy rains have 9.8 percentage points lower of a chance of getting out of poverty. That means that SPEI strongly reduces the urban escalator function of metro core areas.

**Figure 6:**
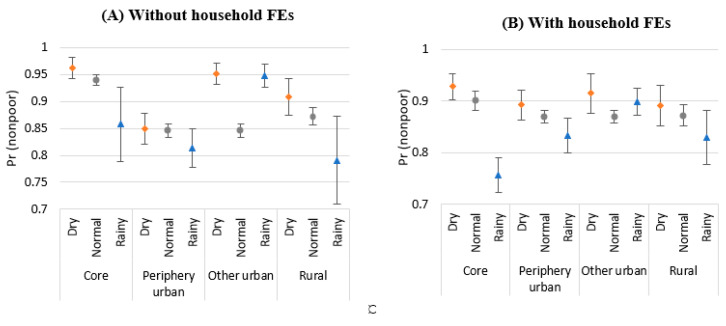
Predicted probability of being nonpoor by locations without household FEs (**A**) and with household FEs (**B**); Source: Authors’ construction.; Note: The predicted probabilities of becoming nonpoor are based on the results of Columns 2 and 4 in Table 5. Error bars indicate 90% CI.

Figure 6 confirms the pattern of SPEI-rainy for metro core areas. Other urban areas with heavy rains show lower predicted probabilities of being nonpoor (with or without household FEs).

Replacing the nonpoor indicator with the status of neither poor nor vulnerable as the outcome variable in Table 9. led to a similar conclusion. The estimated coefficient of the interaction between metro core and SPEI-rainy (Column 4) is 0.131 (90% CI = 0.036), meaning that the chance of being neither poor nor vulnerable for people moving to metro core areas that experience heavy rain decreases by 13.1 percentage points compared with those who did not face heavy rains. The result is less clear for the regressions without household FEs (Column 2).

## 5. Discussion and Conclusions

This paper examines the effects of climatic and environmental shocks on the key function of urban agglomerations in facilitating poverty reduction. Our study showcases different empirical approaches to investigating the association between poverty changes over time and the city population size as well as the heterogeneity of such association by flood risks, depending on the availability of panel data. In particular, we constructed synthetic panel data for Colombia and Chile through repeated cross-sectional household surveys. We further analyzed the probabilities of households escaping poverty in different locations—metro core, urban periphery, other urban areas, and rural areas—and flooding risks in Indonesia, estimating two-way FE models using five waves of IFLS panel data spanning from 1993 to 2015.

The results from the three countries show similar patterns. For Colombia and Chile, we find that the probabilities of households’ transition from poor to nonpoor status were positively correlated with the city population size in both countries. More importantly, such upward mobility was observed only in larger cities with low flood risk. The results of our two-way FE regression analyses for Indonesia suggest that dense metropolitan areas provided good opportunities for migrants to escape poverty. However, high flood risk appears to have reduced such upward mobility in large metropolitan areas. There are several potential reasons. First, heavy rainfall and flooding could impose expensive damage on the dwellings of urban residents. Neighborhoods with high building density and poor infrastructure could be exposed to more damage and thereby higher recovery costs. Second, flooding may lower the productivity and outputs of workers by damaging productive assets, reducing work time, and impeding commuting.

Our findings suggest the importance of reducing flood risks to promote poverty reduction through migration to large metro areas. Upgrading high-density informal settlements would be an effective approach for adaptation. In the Indonesian urbanization context, it would also be important to invest in the peripheries of metropolitan areas, as they have been receiving a large influx of migration. It is essential to reduce congestion forces due to increased migration and better connect peripheries to the cores as the latter provide more poverty-reducing opportunities.

We further clarify some limitations of our study. First, although we employed two-way FE regression models for Indonesia, we could not distinguish the sorting of migrants from the location effects, which would need a stronger identification strategy, such as a natural experimental design. If those with a high capability of escaping from poverty tend to move to cities, our estimates of location effects might be overestimated. Second, we focused on heavy rainfall and flooding as the climatic variable, although other climatic and environmental stressors might undermine the benefits of urban agglomerations as well. 

Finally, this is a case study of three countries at specific time points; thus, we may not be able to generalize the findings to other contexts. We acknowledge that, due to challenges with data access and data harmonization across the countries, the data that we analyzed are not up to date. 

Yet, we believe it is useful to investigate whether climate changes could reduce the role of urbanization in providing economic opportunities for poverty reduction in a multi-country setting. Furthermore, the data covers more or less similar periods, up to the early 2010s for the three countries (Chile during 2011–15, Colombia during 2008–10, Indonesia during 1993/2014–15). We also make new contributions to the literature by demonstrating the usefulness of the empirical approach with and without panel household surveys (with the lack of panel data being a severe data challenge for almost all the poorer countries; see, for example, [31]). 

Hopefully, our paper can motivate future studies to investigate other countries and contribute further insights on the nexus between urbanization, poverty, and climate change. One useful direction is to better analyze new sources of data, such as further expansion of analysis on global poverty at the subnational unit level (see, for example, [39]), to more finely disaggregate the impacts of climate change on urbanization’s beneficial relationship with poverty reduction.

## Figures and Tables

**Table 1:** Summary statistics of Chile and Colombia.

	Count	Mean	SD	Min	Max
Panel A: Chile					
Poor in 2011 (1 = yes, 0 = no)	44,614	0.096	0.295	0.000	1.000
Poor in 2015 (1 = yes, 0 = no)	61,433	0.039	0.193	0.000	1.000
Probability from poor to nonpoor between 2011 and 2015	36,035	0.731	0.101	0.485	0.930
Log of population size in 2015	36,035	10.887	1.334	7.432	14.434
High flood risk (1 = yes, 0 = no)	36,035	0.242	0.429	0.000	1.000
Panel B: Colombia					
Poor in 2008 (1 = yes, 0 = no)	188,801	0.276	0.447	0.000	1.000
Poor in 2010 (1 = yes, 0 = no)	190,344	0.241	0.428	0.000	1.000
Probability from poor to nonpoor between 2008 and 2010	119,692	0.168	0.076	0.053	0.399
Log of population size in 2010	119,692	12.745	0.923	7.988	14.546
High flood risk (1 = yes, 0 = no)	119,692	0.138	0.345	0.000	1.000

Sources: Based on CASEN 2011 and 2015 and GEIH 2008 and 2010. Note: Poverty measure is based on per capita household income, with a threshold of US$5.50 per day. The probability of changing poverty status from poor to nonpoor is estimated based on the synthetic panel approach described in Appendix B). We classify the areas with the top 25 percent flood depth in each country as high flood risk areas (see Section 3.1.3). SD = Standard deviation.

**Table 2:** Summary statistics for Indonesia.

	Count	Mean	SD	Min	Max
Nonpoor (1 = yes, 0 = no)	47,796	0.877	0.328	0.000	1.000
Neither poor nor vulnerable (1 = yes, 0 = no)	47,796	0.690	0.462	0.000	1.000
City: Metro Core (1 = yes, 0 = no)	47,796	0.174	0.379	0.000	1.000
City: Periphery urban (1 = yes, 0 = no)	47,796	0.448	0.497	0.000	1.000
City: Other urban (1 = yes, 0 = no)	47,796	0.192	0.394	0.000	1.000
City: Rural (1 = yes, 0 = no)	47,796	0.186	0.389	0.000	1.000
SPEI: Dry (SPEI < −2) (1 = yes, 0 = no)	47,796	0.059	0.235	0.000	1.000
SPEI: Normal (1 = yes, 0 = no)	47,796	0.907	0.290	0.000	1.000
SPEI: Rainy (SPEI > 2) (1 = yes, 0 = no)	47,796	0.034	0.182	0.000	1.000
High flood risk (1 = yes, 0 = no)	47,796	0.251	0.433	0.000	1.000

Source: Based on IFLS 1993, 1997/98, 2000, 2007/8, and 2014/15. Note: Poverty is measured with the national poverty line; vulnerability is measured with the vulnerability line, which is set at 1.5 times the poverty line. We classify the areas with the top 25 percent flood depth in each country as high flood risk areas (see Section 3.1.3).

**Table 3:** First-difference model (dep var: the probability of transition from poor to nonpoor), Chile.

	(1)	(2)	(3)	(4)
Log population 2015	0.0083 ***		0.0075 ***	0.0089 ***
	(0.0004)		(0.0004)	(0.0004)
Flood risk is high		−0.0174 ***	−0.0136 ***	0.0380 ***
		(0.0012)	(0.0011)	(0.0085)
Flood risk is high # log population 2015				−0.005 ***
				(0.0008)
Constant	0.641 ***	0.736 ***	0.654 ***	0.638 ***
	(0.0041)	(0.0006)	(0.0040)	(0.0050)
Observations	36,035	36,035	36,035	36,035
R-squared	0.012	0.006	0.016	0.016

Note: The table summarizes the estimation results of synthetic panel models in Equation (1). The dependent variable is the probability of each household’s transition from poor to nonpoor between 2011 and 2015. Standard errors in parentheses are estimated at 1000 bootstraps. *** *p* < 0.01.

**Table 4:** First-difference model (dep var: the probability of transition from poor to nonpoor), Colombia.

	(1)	(2)	(3)	(4)
Log of population 2010	0.0029 ***		0.0021 ***	0.0025 ***
	(0.0002)		(0.0002)	(0.0002)
Flood risk is high		−0.0101 ***	−0.009 ***	0.0223 ***
		(0.0006)	(0.0006)	(0.0075)
Flood risk is high # Log pop. 2010				−0.0025 ***
				(0.0006)
Constant	0.132 ***	0.170 ***	0.143 ***	0.138 ***
	(0.0029)	(0.0002)	(0.0030)	(0.0032)
Observations	119,692	119,692	119,692	119,692
R-squared	0.001	0.002	0.003	0.003

Note: The table summarizes the estimation results of synthetic panel models in Equation (1). The dependent variable is the probability of each household’s transition from poor to nonpoor between 2008 and 2010. Standard errors in parentheses are estimated at 1000 bootstraps. *** *p* < 0.01.

**Table 5:** Baseline linear probability models (dep var: nonpoor).

	(1)	(2)	(3)	(4)
City: Core	0.0659 *** (0.0118)	0.0701 *** (0.0119)	0.0145 (0.0207)	0.0310 (0.0193)
City: Periphery urban	−0.0268 ** (0.0125)	−0.0264 ** (0.0125)	−0.0023 (0.0165)	0.0005 (0.0144)
City: Other urban	0.0342 *** (0.0128)	0.0338 *** (0.0128)	0.0054 (0.0250)	0.0073 (0.0225)
City: Rural (Reference)				
Household FE	No	No	Yes	Yes
Year FE	No	Yes	No	Yes
Adjusted R^2^	0.0117	0.0189	−0.0000	0.0058
# of observations	47,795	47,795	47,795	47,795
# of households	18,490	18,490	18,490	18,490

Note: The table summarizes the estimation results of panel regression models in Equation (2) for households in the five waves of IFLS (1993, 1997/8, 2000, 2007/8, and 2014/15). The dependent variable is a binary indicator about household’s poverty status (1 = nonpoor; 0 = poor). Cluster robust standard errors are in parentheses. ** *p* < 0.05, *** *p* < 0.01.

**Table 6:** Baseline linear probability models (dep var: neither poor nor vulnerable).

	(1)	(2)	(3)	(4)
City: Core	0.136 *** (0.0196)	0.141 *** (0.0196)	0.0482 * (0.0286)	0.0701 ** (0.0276)
City: Periphery urban	−0.0504 *** (0.0194)	−0.0503 *** (0.0194)	−0.0168 (0.0260)	−0.0159 (0.0242)
City: Other urban	0.0610 *** (0.0213)	0.0602 *** (0.0212)	0.0310 (0.0271)	0.0325 (0.0254)
City: Rural (reference)				
Household FE	No	No	Yes	Yes
Year FE	No	Yes	No	Yes
Adjusted R^2^	0.0228	0.0308	0.00019	0.0088
# of observations	47,795	47,795	47,795	47,795
# of households	18,490	18,490	18,490	18,490

Note: The table summarizes the estimation results of panel regression models in Equation (2) for households in the five waves of IFLS (1993, 1997/8, 2000, 2007/8, and 2014/15). The dependent variable is a binary indicator about household’s vulnerability status (1 = not vulnerable; 0 = vulnerable). Cluster robust standard errors are in parentheses. * *p* < 0.1, ** *p* < 0.05, *** *p* < 0.01.

**Table 7:** Linear probability models with flood risk (dep var: nonpoor).

	(1)	(2)	(3)	(4)
City: Core	0.0634 *** (0.0123)	0.0601 *** (0.0149)	0.0293 (0.0194)	0.0467 ** (0.0196)
City: Periphery urban	−0.0274 ** (0.0124)	−0.0299 * (0.0157)	−0.00034 (0.0143)	0.0176 (0.0157)
City: Other urban	0.0304 ** (0.0127)	0.0349 ** (0.0153)	0.0064 (0.0227)	0.0189 (0.0231)
City: Rural (reference)				
High flood risk	−0.0257 *** (0.0092)	−0.0282 (0.0192)	−0.0107 (0.0111)	0.0317 * (0.0189)
City: Core # High flood risk		0.0329 (0.0253)		−0.0743 * (0.0404)
City: Periphery urban # High flood risk		0.00827 (0.0235)		−0.0533 ** (0.0244)
City: Other urban # High flood risk		−0.0232 (0.0270)		−0.0322 (0.0259)
Household FE	No	No	Yes	Yes
Year FE	Yes	Yes	Yes	Yes
Adjusted R^2^	0.0200	0.0203	0.0058	0.0061
# of observations	47,795	47,795	47,795	47,795
# of households	18,490	18,490	18,490	18,490

Note: The table summarizes the estimation results of panel regression models in Equation (3) for households in the five waves of IFLS (1993, 1997/8, 2000, 2007/8, and 2014/15). The dependent variable is a binary indicator about household’s poverty status (1 = nonpoor; 0 = poor). Cluster robust standard errors are in parentheses. * *p* < 0.1, ** *p* < 0.05, *** *p* < 0.01.

**Table 8:** Linear probability models with SPEI (dep var: nonpoor).

	(1)	(2)	(3)	(4)
City: Core	0.0676 *** (0.0115)	0.0670 *** (0.0112)	0.0272 (0.0197)	0.0273 (0.0197)
City: Periphery urban	−0.0263 ** (0.0124)	−0.0268 ** (0.0118)	−0.0025 (0.0151)	−0.0032 (0.0152)
City: Other urban	0.0323 ** (0.0125)	0.0284 ** (0.0123)	0.0047 (0.0233)	0.0036 (0.0233)
City: Rural (reference)				
SPEI: Dry	0.0220 ** (0.0104)	0.0358 * (0.0212)	0.0256 ** (0.0109)	0.0185 (0.0200)
SPEI: Normal (reference)				
SPEI: Rainy	−0.0406 ** (0.0193)	−0.0817 * (0.0460)	−0.0388 ** (0.0155)	−0.0445 (0.0310)
City: Core # SPEI: dry		−0.0135 (0.0215)		0.0090 (0.0198)
City: Core # SPEI: rainy		−0.00019 (0.0619)		−0.0985 *** (0.0356)
City: Periphery urban # SPEI: dry		−0.0323 (0.0259)		0.0039 (0.0252)
City: Periphery urban # SPEI: rainy		0.0495 (0.0496)		0.0078 (0.0363)
City: Other urban # SPEI: dry		0.0141 (0.0226)		0.0193 (0.0257)
City: Other urban # SPEI: rainy		0.128 *** (0.0473)		0.0657 ** (0.0317)
Household FE	No	No	Yes	Yes
Year FE	Yes	Yes	Yes	Yes
Adjusted R^2^	0.0195	0.0199	0.0065	0.0068
# of observations	47,795	47,795	47,795	47,795
# of households	18,490	18,490	18,490	18,490

Note: The table summarizes the estimation results of panel regression models in Equation (3) for households in the five waves of IFLS (1993, 1997/8, 2000, 2007/8, and 2014/15). The dependent variable is a binary indicator about household’s poverty status (1 = nonpoor; 0 = poor). Cluster robust standard errors are in parentheses. * *p* < 0.1, ** *p* < 0.05, *** *p* < 0.01.

**Table 9:** Linear probability model, regression with SPEI (dep var: neither poor nor vulnerable).

	(1)	(2)	(3)	(4)
City: Core	0.137 *** (0.0193)	0.134 *** (0.0193)	0.0646 ** (0.0284)	0.0637 ** (0.0283)
City: Periphery urban	−0.0504 *** (0.0192)	−0.0502 *** (0.0190)	−0.0207 (0.0256)	−0.0199 (0.0255)
City: Other urban	0.0581 *** (0.0210)	0.0556 *** (0.0214)	0.0285 (0.0265)	0.0281 (0.0264)
City: Rural (reference)				
SPEI: Dry	0.0145 (0.0181)	0.0235 (0.0316)	0.0273 * (0.0155)	0.00452 (0.0271)
SPEI: Normal (reference)				
SPEI: Rainy	−0.0610 ** (0.0243)	−0.0900 ** (0.0419)	−0.0598 *** (0.0186)	−0.0229 (0.0204)
City: Core # SPEI: Dry		0.0195 (0.0348)		0.0433 (0.0293)
City: Core # SPEI: Rainy		0.0266 (0.0690)		−0.131 *** (0.0362)
City: Periphery urban # SPEI: Dry		−0.0369 (0.0407)		0.0150 (0.0354)
City: Periphery urban # SPEI: Rainy		0.0359 (0.0495)		−0.0434 (0.0314)
City: Other urban # SPEI: Dry		0.0132 (0.0384)		0.0373 (0.0423)
City: Other urban # SPEI: Rainy		0.0558 (0.0768)		0.0541 (0.0370)
Household FE	No	No	Yes	Yes
Year FE	Yes	Yes	Yes	Yes
Adjusted R^2^	0.0312	0.0313	0.0097	0.0099
# of observations	47,795	47,795	47,795	47,795
# of households	18,490	18,490	18,490	18,490

Note: The table summarizes the estimation results of panel regression models in Equation (3) for households in the five waves of IFLS (1993, 1997/8, 2000, 2007/8, and 2014/15). The dependent variable is a binary indicator about household’s vulnerability status (1 = not vulnerable; 0 = vulnerable). Cluster robust standard errors are in parentheses * *p* < 0.1, ** *p* < 0.05, *** *p* < 0.01.

## Data Availability

The data are obtained in confidential terms, but we will provide contact information on how to access the data.

## Appendix A. Additional Tables

**Table A1:** Summary statistics of Chile (2011–2015).

			Difference
	2011	2015	
The logarithm of per capita income	5.875	6.177	0.302 ***
	(0.039)	(0.034)	(0.016)
Head’s age	42.940	45.607	2.667
	(0.111)	(0.121)	(0.153)
Head is female	0.342	0.340	−0.002
	(0.008)	(0.005)	(0.009)
Head does not complete primary school	0.133	0.118	−0.015 ***
	(0.007)	(0.006)	(0.004)
Head’s highest education level is primary	0.300	0.260	−0.039 ***
	(0.010)	(0.008)	(0.007)
Head’s highest education level is secondary	0.397	0.410	0.013
	(0.013)	(0.010)	(0.008)
Head’s highest education level is tertiary	0.170	0.211	0.041 ***
	(0.018)	(0.016)	(0.008)
Urban area	0.879	0.875	−0.004
	(0.012)	(0.012)	(0.004)

Note: Standard errors are in parentheses, and the differences are estimated, considering the complex survey design. *** *p* < 0.01. Population weights are applied. We do not test for the difference in the distributions of the age variable in the two survey rounds since it is a deterministic variable. Household heads’ ages are restricted to between 25 and 55 for the first survey round and between 29 and 59 for the second survey round.

**Table A2:** Summary statistics of Colombia (2008–2010).

			Difference
	2008	2010	
The logarithm of per capita income	5.341	5.422	0.081 ***
	(0.052)	(0.044)	(0.020)
Head’s age	41.073	42.317	1.243
	(0.104)	(0.075)	(0.070)
Head is female	0.261	0.285	0.023 ***
	(0.008)	(0.008)	(0.004)
Head does not complete primary school	0.225	0.239	0.014 *
	(0.016)	(0.017)	(0.008)
Head’s highest education level is primary	0.385	0.372	−0.013 ***
	(0.004)	(0.005)	(0.005)
Head’s highest education level is secondary	0.288	0.293	0.004
	(0.010)	(0.011)	(0.005)
Head’s highest education level is tertiary	0.101	0.097	−0.005
	(0.007)	(0.007)	(0.003)
Urban area	0.814	0.815	0.002
	(0.031)	(0.028)	(0.011)

Note: Standard errors are in parentheses, and the differences are estimated, considering the complex survey design. * *p* < 0.1, *** *p* < 0.01. Population weights are applied. We do not test for the difference in the distributions of the age variable in the two survey rounds since it is a deterministic variable. Household heads’ ages are restricted to between 25 and 55 for the first survey round and between 27 and 57 for the second survey round.

**Table A3:** Estimated OLS model of household income per capita in 2015, Chile.

	Coef/SE
Head’s age	0.014 ***
	(0.00)
=1 if the head is female	−0.139 ***
	(0.01)
Education level (reference—if head does not complete primary school)	
=1 if the head’s highest education level is primary	0.129 ***
	(0.01)
=1 if the head’s highest education level is secondary	0.398 ***
	(0.02)
=1 if the head’s highest education level is tertiary	1.183 ***
	(0.07)
=1 if the area of residence is urban	0.016
	(0.02)
Constant	5.141 ***
	(0.04)
Adjusted R^2^	0.294
Number of observations	45,954

Note: Standard errors clustered at primary sampling units are in parentheses, *** *p* < 0.01. The dependent variable is the logarithm of household income per capita. Household heads’ ages are restricted to between 29 and 59.

**Table A4:** Estimated OLS model of household income per capita in 2010, Colombia.

	Coef/SE
Head’s age	0.019 ***
	(0.00)
=1 if the head is female	−0.127 ***
	(0.02)
Education level (reference—if head does not complete primary school)	
=1 if the head’s highest education level is primary	0.319 ***
	(0.02)
=1 if the head’s highest education level is secondary	0.767 ***
	(0.02)
=1 if the head’s highest education level is tertiary	1.704 ***
	(0.05)
=1 if the area of residence is urban	0.358 ***
	(0.06)
Constant	3.915 ***
	(0.05)
Adjusted R^2^	0.348
Number of observations	133,483

Note: Standard errors clustered at primary sampling units are in parentheses. *** *p* < 0.01. The dependent variable is the logarithm of household income per capita. Household heads’ ages are restricted to between 30 and 60.

**Table A5:** Residential movement across IFLS waves, Indonesia (percentage of household).

		Metro Core	Periphery U	Other U	Rural
		Wave 2			
Wave 1	Metro core	95.2	0.7	3.5	0.6
	Periphery U	0.1	99.4	0.2	0.3
	Other U	0.3	0.3	99.3	0.2
	Rural	0.0	0.7	0.3	99.0
		Wave 3			
		Metro core	Periphery U	Other U	Rural
Wave 2	Metro core	96.2	1.6	2.0	0.3
	Periphery U	0.2	95.7	1.2	3.0
	Other U	0.6	4.0	95.0	0.3
	Rural	0.1	13.9	2.9	83.2
		Wave 4			
		Metro core	Periphery U	Other U	Rural
Wave 3	Metro core	91.9	2.2	4.9	1.1
	Periphery U	0.3	93.1	2.2	4.4
	Other U	1.6	3.3	91.0	4.2
	Rural	0.4	8.9	0.8	89.9
		Wave 5			
		Metro core	Periphery U	Other U	Rural
Wave 4	Metro core	91.8	2.5	4.5	1.2
	Periphery U	0.3	98.6	0.4	0.7
	Other U	2.4	1.5	95.5	0.7
	Rural	0.7	2.0	0.7	96.7

## Appendix B. Synthetic Panel Method

This appendix provides a summary of the synthetic panel method based on [29,30]. Let $x_{\mathrm{ij}}$ be a vector of household characteristics observed in survey round j (j = 1 or 2) that are also observed in the other survey round for household i, i = 1…, N. These household characteristics can include such time-invariant variables as ethnicity, religion, language, place of birth, parental education, and other time-varying household characteristics if retrospective questions about the round-1 values of such characteristics are asked in the second-round survey. To reduce spurious changes due to changes in household composition over time, we usually restrict the estimation samples to household heads in a certain age range, say 25 to 55, in the first cross-section and adjust this age range accordingly in the second cross-section. This restriction also helps to ensure that certain variables, such as heads’ education attainment, remain relatively stable over time (assuming that most heads in the given age range are finished with their schooling). This age range is usually used in traditional pseudo-panel analysis, but can vary depending on the cultural and economic factors in each specific setting. Population weights are then employed to provide estimates that represent the whole population.

Then let $y_{\mathrm{ij}}$ represent household consumption or income in survey round j, j = 1 or 2. The linear projection of household consumption (or income) on household characteristics for each survey round is given by

(A1) $$y_{\mathrm{ij}}=\beta_{j}^{\prime }x_{\mathrm{ij}}+\varepsilon_{\mathrm{ij}}$$

Let $z_{j}$ be the poverty line in period j. We are interested in knowing the unconditional measures of poverty mobility such as

(A2) $$P\left(y_{i1}<z_{1}{\;\mathrm{and}\;y}_{i2}>z_{2}\right)$$

which represents the percentage of households that are poor in the first survey round (year) but nonpoor in the second survey round, or the conditional measures such as

(A3) $$P\left(y_{i2}>z_{2}|{\;y}_{i1}<z_{1}\right)$$

which represents the percentage of poor households in the first round that escape poverty in the second round.

If true panel data are available, we can straightforwardly estimate the quantities in (2) and (3); in the absence of such data, we can use synthetic panels to study mobility. To operationalize the framework, we make two standard assumptions. First, we assume that the underlying population being sampled in the first and second survey rounds are identical such that their time-invariant characteristics remain the same over time. More specifically, coupled with equation (1), this implies that the conditional distribution of expenditure in a given period is identical whether it is conditional on the given household characteristics in the first period or the second period (i.e., $x_{i1}=x_{i2}$ implies $y_{i1}|x_{i1}$ and $y_{i1}|x_{i2}$ have identical distributions). Second, we assume that $\varepsilon_{i1}$ and $\varepsilon_{i2}\;$have a bivariate normal distribution with positive correlation coefficient ρ and standard deviations $\sigma_{\epsilon_{1}}\;$and $\sigma_{\epsilon_{2}}$ respectively. Quantity (2) can be estimated by

(A4) $$P\left(y_{i1}<z_{1}{\;\mathrm{and}\;y}_{i2}>z_{2}\right)=\Phi_{2}\left(\frac{z_{1}-\beta_{1}^{\prime }x_{i2}}{\sigma_{\varepsilon_{1}}},-\frac{z_{2}-\beta_{2}^{\prime }x_{i2}}{\sigma_{\varepsilon_{2}}},-\rho \right)$$

where $\Phi_{2}\left(.\right)$ stands for the bivariate normal cumulative distribution function (cdf)) (and $\phi_{2}\left(.\right)$ stands for the bivariate normal probability density function (pdf)). Note that in Equations (1) and (A1), the estimated parameters obtained from data in both survey rounds are applied to data from the second survey round (x_2_) (or the base year) for prediction, but we can use data from the first survey round as the base year as well. It is then straightforward to estimate quantity (3) by dividing quantity (2) by $\Phi \left(\frac{z_{1}-\beta_{1}^{\prime }x_{i2}}{\sigma_{\varepsilon_{1}}}\right)$, where $\Phi \left(.\right)$ stands for the univariate normal cumulative distribution function (cdf).

In Equation (A1), the parameters $\beta_{j}$ and $\sigma_{\varepsilon_{j}}$ are estimated from Equation (1) and ρ can be estimated using an approximation of the correlation of the cohort-aggregated household consumption between the two surveys ($\rho_{y_{c1}y_{c2}}$). Given an approximation of $\rho_{y_{c1}y_{c2}}$, where c indexes the cohorts constructed from the household survey data, the partial correlation coefficient ρ can be estimated by

(A5) $$\rho =\frac{\rho_{y_{i1}y_{i2}}\sqrt{\mathrm{var}\left(y_{i1}\right)\mathrm{var}\left(y_{i2}\right)}-\beta_{1}^{\prime }\mathrm{var}\left(x_{i}\right)\beta_{2}}{\sigma_{\varepsilon_{1}}\sigma_{\varepsilon_{2}}}$$

An alternative way to estimate ρ is to aggregate all the time-invariant variables to the cohort level and use the following equation

(A6) $$y_{\mathrm{cj}}=\beta_{j}^{\prime }x_{\mathrm{cj}}+\varepsilon_{\mathrm{cj}}$$

where the error term $\varepsilon_{\mathrm{cj}}$ includes a cohort fixed effect $\tau_{c}$ and the error $\nu_{\mathrm{cj}}$.

Note that the standard errors of estimates based on the synthetic panels can in fact be even smaller than that of the true (or design-based) rate if there is a good model fit (or the sample size in the target survey is significantly larger than that in the base survey; see [30] for more discussion).

## References

[1] United Nations (2019). World Urbanization Prospects: The 2018 Revision (ST/ESA/SER.A/420).

[2] Duranton G., Puga D. (2004). Micro-foundations of Urban Agglomeration Economies. Handbook of Regional and Urban Economics.

[3] Glaeser E.L., Kallal H.D., Scheinkman J.A., Shleifer A. (1992). Growth in Cities. J. Political Econ..

[4] Glaeser E.L., Gottlieb J.D. (2009). The Wealth of Cities: Agglomeration Economies and Spatial Equilibrium in the United States. J. Econ. Lit..

[5] Michaels G., Rauch F., Redding S.J. (2012). Urbanization and Structural Transformation. Q. J. Econ..

[6] Hallegatte S., Vogt-Schilb A., Bangalore M., Rozenberg J. (2017). Unbreakable: Building the Resilience of the Poor in the Face of Natural Disasters.

[7] Mukim M., Roberts M. (2022). Thriving: Making Cities Green, Resilient, and Inclusive in a Challenging Climate.

[8] Lagakos D. (2020). Urban-Rural Gaps in the Developing World: Does Internal Migration Offer Opportunities?. J. Econ. Perspect..

[9] Beegle K., De Weerdt J., Dercon S. (2011). Migration and Economic Mobility in Tanzania: Evidence from a Tracking Survey. Rev. Econ. Stat..

[10] Bryan G., Chowdhury S., Mobarak A. (2014). Underinvestment in a Profitable Technology: The Case of Seasonal Migration in Bangladesh. Econometrica.

[11] Combes P.-P., Demurger S., Li S., Wang J. (2020). Unequal Migration and Urbanisation Gains in China. J. Dev. Econ..

[12] Hamory J., Kleemans M., Li N.Y., Miguel E. (2021). Reevaluating Agricultural Productivity Gaps with Longitudinal Microdata. J. Eur. Econ. Assoc..

[13] Glaeser E., Maré D. (2001). Cities and Skills. J. Labor Econ..

[14] Melo P.C., Graham D.J., Noland R.B. (2009). A Meta-Analysis of Estimates of Urban Agglomeration Economies. Reg. Sci. Urban Econ..

[15] Puga D. (2010). The Magnitude and Causes of Agglomeration Economies. J. Reg. Sci..

[16] Rosenthal S.S., Strange W.C., Henderson J.V., Thisse J.F. (2004). Evidence on the Nature and Sources of Agglomeration Economies. Handbook of Regional and Urban Economics.

[17] Chauvin J., Glaeser E., Ma Y., Tobio K. (2017). What Is Different about Urbanization in Rich and Poor Countries? Cities in Brazil, China, India and the United States. J. Urban Econ..

[18] Combes P.-P., Nakamura S., Roberts M., Stewart B. Estimating Urban Poverty Consistently Across Countries. World Bank Poverty and Equity Notes 2022, 48. https://openknowledge.worldbank.org/handle/10986/37698.

[19] Grover A., Lall S.V., Timmis J. Agglomeration Economies in Developing Countries: A Meta-Analysis. World Bank Policy Research Working Paper 2021, 9730. https://openknowledge.worldbank.org/handle/10986/36003.

[20] Quintero L.E., Roberts M. Cities and Productivity: Evidence from 16 Latin American and Caribbean Countries. Johns Hopkins Carey Business School Research Paper 2022, 22-13. https://papers.ssrn.com/sol3/papers.cfm?abstract_id=4106477.

[21] Bloom D.E., Canning D., Fink G. (2008). Urbanization and the Wealth of Nations. Science.

[22] Castells-Quintana D., Wenban-Smith H. (2020). Population Dynamics, Urbanisation without Growth, and the Rise of Megacities. J. Dev. Stud..

[23] Fay M., Opal C. (1999). Urbanization without Growth: A Not-So-Uncommon Phenomenon.

[24] Gollin D., Jedwab R., Vollrath D. (2016). Urbanization with and without industrialization. J. Econ. Growth.

[25] Dijkstra L., Florczyk A.J., Kemper T., Melchiorri M., Pesaresi M., Shiavina M. (2021). Applying the degree of urbanisation to the globe: A new harmonised definition reveals a different picture of global urbanisation. J. Urban Econ..

[26] Gibson J., Jiang Y., Susantono B. Revisiting the Role of Secondary Towns: Effects of Different Types of Urban Growth on Poverty in Indonesia. University of Waikato Working Paper in Economics 2022, 5/22. https://repec.its.waikato.ac.nz/wai/econwp/2205.pdf.

[27] Christiaensen L., Todo Y. (2014). Poverty Reduction during the Rural-Urban Transformation: The Role of the Missing Middle. World Dev..

[28] Baez J.E., Lucchetti L., Genoni M.E., Salazar M. (2017). Gone with the Storm: Rainfall Shocks and Household Wellbeing in Guatemala. J. Dev. Stud..

[29] Dang H.-A., Lanjouw P., Luoto J., McKensize D. (2014). Using Repeated Cross-Sections to Explore Movements into and out of Poverty. J. Dev. Econ..

[30] Dang H.-A., Lanjouw P. (2023). Measuring Poverty Dynamics with Synthetic Panels Based on Cross-Sections. Oxf. Bull. Econ. Stat..

[31] Dang H.-A., Jolliffe D., Carletto C. (2019). Data Gaps, Data Incomparability, and Data Imputation: A Review of Poverty Measurement Methods for Data-Scarce Environments. J. Econ. Surv..

[32] Dang H.-A., Lanjouw P., Silber J. (2023). Regression-Based Imputation for Poverty Measurement in Data Scarce Settings. Research handbook on Measuring Poverty and Deprivation.

[33] Garcés-Urzainqui D., Lanjouw P., Rongen G. (2021). Constructing Synthetic Panels for the Purpose of Studying Poverty Dynamics: A Primer. Rev. Dev. Econ..

[34] Setiawan I., Tiwari S., Rizal H. (2018). Economic and Social Mobility in Urbanizing Indonesia 2018. Personal communication.

[35] Roberts M., Sander F.G., Tiwari S. (2019). Time to Act: Realizing Indonesia’s Urban Potential.

[36] Duranton G. (2015). A Proposal to Delineate Metropolitan Areas in Colombia. Revista Desarrollo y Sociedad.

[37] Vicente-Serrano S.M., Beguería S., Lopez-Moreno J.I. (2010). A Multiscalar Drought Index Sensitive to Global Warming: The Standardized Precipitation Evapotranspiration Index. J. Clim..

[38] Harari M., Ferrara E.L. (2018). Conflict, Climate, and Cells: A Disaggregated Analysis. Rev. Econ. Stat..

[39] Dang H.A.H., Nguyen M.C., Trinh T.A. Does Hotter Temperature Increase Poverty? Global Evidence from Subnational Data Analysis 2022. ECINEQ Working Paper 622. http://www.ecineq.org/2022/09/23/does-hotter-temperature-increase-poverty-global-evidence-from-subnational-data-analysis/.

